# Designing AI-resilient assessment in higher education: a four-pillar conceptual framework

**DOI:** 10.3389/frai.2026.1841682

**Published:** 2026-07-16

**Authors:** Dragan Nikolić, Marijana Basta Nikolić

**Affiliations:** Faculty of Medicine, University of Novi Sad, Novi Sad, Serbia

**Keywords:** AI literacy, AI-resilient assessment, authentic assessment, generative artificial intelligence, higher education assessment, medical education, oral defense

## Abstract

Generative AI tools can produce polished academic text on demand, undermining the validity of assessments that treat written submissions as evidence of individual learning. Detection-based countermeasures have demonstrated variable accuracy and equity concerns. This paper does not report empirical outcomes or validation data. It proposes a framework for AI-resilient assessment that shifts evaluation from product quality to demonstrable reasoning, decision-making, and ownership of learning. The framework comprises four pillars: (1) process-based documentation; (2) oral defense integration; (3) authentic task design; and (4) transparent AI-use policies aligned with intended learning outcomes. These are operationalized through a rubric model, an oral-defense protocol, and an assessment vulnerability audit tool. Illustrated primarily through health sciences education, with wider relevance to professional and practice-oriented disciplines, the framework treats generative AI as a catalyst for re-examining how assessment evidence is generated rather than solely as an integrity threat. All proposed tools are design instruments for future empirical evaluation, not psychometrically validated instruments.

## 1 Introduction

Many early institutional responses to generative AI have emphasized detection tools and misconduct procedures, often more than assessment redesign (Weber-Wulff et al., 2023). Independent evaluations have exposed critical limitations: detection accuracy varies across writing styles and languages; false-positive rates disproportionately affect non-native English speakers; and detection–evasion cycles may foster surveillance rather than trust (Liang et al., 2023; Elkhatat et al., 2023). More fundamentally, detection addresses unauthorized AI use without confronting whether the assessment itself measures what educators value in graduates (Bearman et al., 2023; Cotton et al., 2024).

In professional education, a student may construct an exemplary case summary with AI assistance yet remain unable to justify a treatment decision under uncertainty (Kung et al., 2023). Such scenarios reveal a misalignment between what the assessment measures and what the programme expects graduates to demonstrate—what we term the *assessment validity gap* (Corbin et al., 2025a). Product-focused assessments may privilege surface quality over authentic reasoning—a limitation that generative AI has made more visible.

This conceptual analysis proposes a framework that redirects evaluation from product quality to the student’s capacity to demonstrate understanding through explanation, application, and real-time reasoning. The framework integrates four pillars—process documentation, oral defense, authentic task design, and transparent AI-use policies aligned with intended learning outcomes (ILOs)—operationalized through practical design tools. The derivation logic is described in Section 5.1.

### 1.1 Conceptual approach and scope

This manuscript is a conceptual analysis, not an empirical study or systematic review. The framework is informed by a focused synthesis of peer-reviewed scholarship across five domains: assessment validity and design (including Messick, 1989; Biggs and Tang, 2011), authentic assessment, oral and interactive assessment, generative AI in higher education, and AI-detection limitations. Sources were identified through searches in Scopus, Web of Science, and Google Scholar (January 2020–March 2026) using search strings combining “generative AI” OR “large language model” with “assessment,” “academic integrity,” “oral defense,” and “authentic assessment.” Backward citation tracking of key publications supplemented the search. Because this is a conceptual analysis rather than a scoping review, the search was used to identify representative theoretical and policy-oriented approaches rather than to exhaustively capture all publications. Sources were included if they: (a) addressed assessment design, validity, or integrity in the context of generative AI or automated text production; (b) were published in peer-reviewed journals, edited volumes, or as institutional policy documents from recognized higher education bodies; and (c) provided theoretical analysis, empirical evidence, or operational recommendations relevant to at least one of the four focal dimensions. Sources were excluded if they focused exclusively on AI-detection tool performance without addressing assessment design implications, or if they addressed AI in education without relevance to assessment validity.

Frameworks included in the comparison table (Table 1) were selected if they: (a) explicitly addressed assessment redesign in response to generative AI, (b) were published in 2024–2026, and (c) provided sufficiently detailed descriptions of at least one of the four focal dimensions. Components were coded as present (✓), partially addressed (Partial), or absent (−) based on whether the source explicitly described the component as a design recommendation with operational detail.

**Table 1:** Comparison with existing AI-resilient assessment proposals.

Framework	Source	Process	Oral	Auth.	Policy	Operationalization Level	What is absent
Awadallah Alkouk and Khlaif (2024)	Peer-rev.	✓	✓	✓	Partial	General principles	No rubric, protocol, or audit tool; validity-theory grounding not made explicit.
Corbin et al. (2025b)	Peer-rev.	Partial	—	✓	✓	Specific recommendations	No oral-defense component; no transferable operational tools; no explicit vulnerability analysis.
University of Sydney (2025)	Institutional	—	✓	✓	✓	Institutional guidance	No process-documentation component; no rubric model; cross-disciplinary adaptation logic not explicitly developed.
Higher Education Authority (HEA) (2025)	Policy	✓	Partial	✓	✓	National policy	No rubric or protocol; no explicit vulnerability analysis; not developed at discipline level.
This manuscript	This ms.	✓	✓	✓	✓	Transferable tools	Integrates all four pillars; provides rubric, protocol, taxonomy, audit, and vulnerability analysis.

Coding: ✓ = component explicitly described with operational detail; Partial = mentioned but not operationalized; — = absent. Both authors independently coded all entries; initial agreement was 14/16 decisions (87.5%); disagreements resolved through discussion. The present framework was self-coded, which represents a potential bias. The table includes both peer-reviewed papers and institutional/policy frameworks, which differ in scope and purpose.

All proposed tools—rubric, protocol, taxonomy, audit tool—are design instruments for future evaluation, not psychometrically validated instruments. All AI-assisted outputs were reviewed and verified by the authors; the AI tool was not used to generate original references or unverified claims (Table 2).

**Table 2:** Definitions, boundaries, and assessment implications of key concepts.

Concept	Definition	Distinguished from	Assessment implication
AI-resilient assessment	Assessment designed so that valid evidence of learning is not substantially diminished by student AI access.	AI-resistant (unattainable); AI-proof.	Design should strengthen evidence channels, not merely restrict tools.
Ownership of learning	Demonstrable capacity to explain, justify, and extend reasoning behind submitted work.	Authorship verification; plagiarism detection.	Oral defense and process logs provide stronger evidence than product alone.
Assessment validity gap	Misalignment between what an assessment format measures and what learning outcomes require.	Academic misconduct (behavioral); detection failure (technical).	Requires design-level solutions, not post hoc verification.

## 2 The assessment validity gap

### 2.1 Limitations of AI detection tools

AI-detection tools analyze statistical text features to estimate the probability of machine authorship (Mitchell et al., 2023). Weber-Wulff et al. (2023) evaluated fourteen tools; notably, GPTZero and ZeroGPT produced false-positive rates exceeding 20% on specific samples, while others performed near chance on paraphrased AI text. Liang et al. (2023) demonstrated systematic misclassification of non-native English writing. As detection improves, evasion co-evolves: paraphrasing and prompt engineering can render AI text effectively undetectable (Krishna et al., 2023). Current evidence therefore suggests these tools remain inconsistent and may introduce fairness concerns when used as primary integrity evidence.

### 2.2 Pedagogical limitations of detection-centered assessment

Detection does not address whether the assessment validly captures what students know and can do. When a language model can produce a passing submission without meaningful learner engagement, the assessment’s construct validity is compromised (Corbin et al., 2025a; Dawson et al., 2024; Lodge et al., 2023).

### 2.3 Toward design-centered assessment

These limitations suggest that the primary response should shift from *post hoc* detection toward assessment designs that require students to demonstrate reasoning and application (Sadler, 2009). Detection tools may retain utility as one signal, but the assessment architecture itself should bear primary responsibility for generating valid evidence of competence.

## 3 Relation to existing frameworks

Several recent proposals address AI-resilient assessment with overlapping components. Table 1 provides a structured comparison. The table includes both peer-reviewed conceptual papers and institutional/policy frameworks, as noted in the source-type column.

### 3.1 What this framework adds

The contribution of this framework is not limited to integrating components that appear individually in prior work. Three dimensions distinguish it from existing proposals.

First, theoretically grounded pillar derivation. Existing frameworks assemble assessment strategies inductively from observed good practice. The present framework derives its four pillars deductively from Messick’s (1989) unified validity framework, mapping each pillar to a specific validity threat created by generative AI (see Section 5.1). This ensures that the pillars are jointly necessary and individually justified, not merely a convenient grouping.

Second, operational specificity. Existing proposals typically stop at the level of general principles or recommendations. This framework provides transferable tools: a rubric model with worked scoring examples (Table 3), a structured oral-defense protocol with time estimates and workload analysis (Section 6.2), a graduated AI-use taxonomy with disclosure requirements (Table 4), and a vulnerability audit tool (Table 5). These instruments are designed to be immediately usable, subject to local adaptation and future validation.

**Table 3:** Rubric model for AI-resilient assessment.

Criterion (Weight Range)	Excellent (4)	Proficient (3)	Developing (2)	Inadequate (1)
Reasoning quality (25–35%)	Nuanced reasoning. Anticipates counterarguments.	Clear reasoning; some alternatives considered.	Superficial or inconsistent.	Absent or incoherent.
Oral defense (25–35%)	Articulates fluently. Extends to novel scenarios.	Explains clearly. Some transfer.	Basic; struggles with probing.	Unable to explain submitted work.
Process engagement (20–30%)	Genuine intellectual trajectory visible.	Shows progression and revision.	Limited process evidence.	No process documentation.
Communication/product quality (10–20%)	Well-structured within disciplinary conventions.	Adequate; minor errors.	Weaknesses impeding clarity.	Poorly structured.

Weights are approximate ranges; not all combinations within these ranges are valid. Selected weights must sum to exactly 100%. Programs select one value per criterion before implementation. Worked example: Reasoning 30% + Oral Defense 30% + Process 25% + Product 15% = 100%. A student scoring Excellent (4) on Reasoning, Proficient (3) on Oral Defense, Proficient (3) on Process, and Excellent (4) on Product: Total = (4 × 0.30) + (3 × 0.30) + (3 × 0.25) + (4 × 0.15) = 1.20 + 0.90 + 0.75 + 0.60 = 3.45/4.00.

Accommodation note: For students with documented communication disabilities, the oral-defense weight may be partially redistributed to process engagement or replaced with alternative verification modes (written viva, asynchronous video). Programs should document accommodation decisions.

**Table 4:** Graduated AI-use policy taxonomy.

Level	Description	Example tasks	Disclosure	Illustrative ILOs
Prohibited	No AI. Independent competency required.	Timed in-class essay; OSCE; closed-book exam.	None needed.	Compose original reasoning independently.
Guided	AI for delimited tasks. Student documents and critiques AI use.	Annotated portfolio with AI-use log; student-authored synthesis.	AI-use log; prompts disclosed.	Critically evaluate AI output.
Permitted	AI fully permitted. Assessment targets higher-order competencies.	AI analysis + student critique + oral defense.	AI use acknowledged.	Apply professional judgment to AI outputs.

ILO = intended learning outcome; OSCE = objective structured clinical examination; LLM = large language model. Examples are illustrative. “Permitted” does not mean absence of accountability; it shifts evaluation to critique and judgment.

**Table 5:** Assessment vulnerability audit tool.

Audit question	Vulnerability	Pillar(s)	Typical redesign action
Could a competent LLM user pass without engaging the learning material?	High	Authentic tasks; Oral defense	Add oral defense; embed contextual data.
Is only the final product evaluated?	High	Process documentation	Require staged submissions, revision logs.
Is the task generic enough for any student’s prompt?	High	Authentic tasks; Policy	Personalize; add context-specific constraints.
Does the rubric emphasize polish over reasoning?	Moderate	Rubric redesign	Reweight toward reasoning and process.
Does the AI-use policy specify ILO-aligned expectations?	High (if no)	Policy (Table 4)	Develop graduated taxonomy with disclosure.
Does the assessment include synchronous oral verification of understanding?	Low (if yes)	Oral defense already present	Maintain; verify standardization and accessibility.

Vulnerability levels are heuristic categories based on the authors’ conceptual analysis, not validated thresholds. They should be determined through local program-level review.

Third, self-critical vulnerability analysis. No existing framework systematically examines how its own components might be circumvented by evolving AI capabilities. Section 9.5 provides this analysis, including second-order safeguards, explicitly acknowledging that no assessment design is permanently invulnerable.

More specifically, the framework maps each validity threat to a corresponding evidentiary gap and design response:

- Invisible process (threat) → no evidence of reasoning trajectory (gap) → Process Documentation (response).
- Pre-prepared output (threat) → no evidence of real-time understanding (gap) → Oral Defense (response).
- Generic task (threat) → no evidence of situated judgment (gap) → Authentic Task Design (response).
- Ambiguous expectations (threat) → no normative clarity (gap) → AI-Use Policy (response).

## 4 Key concepts and definitions

The term *AI-resilient* is used deliberately in preference to *AI-resistant* (cf. Awadallah Alkouk and Khlaif, 2024). “Resistant” implies impermeability—an unattainable standard. “Resilient” denotes an assessment whose capacity to generate valid evidence of learning is maintained even when students have access to AI. This aligns with Messick’s (1989) emphasis on construct validity: the question is whether the assessment evidences the targeted construct, not whether it excludes particular tools.

To move beyond a purely normative definition, Table 6 proposes operational indicators by which the resilience of an assessment design can be evaluated. These indicators are starting points for empirical investigation; future research should examine whether assessments scoring higher on these indicators are indeed less susceptible to AI-assisted completion without genuine learning. These indicators should be interpreted as provisional and context-sensitive, with their adequacy depending on disciplinary aims, assessment stakes, and local implementation constraints.

**Table 6:** Operational indicators of assessment resilience.

Pillar	Operational indicator	How to assess
Process documentation	≥2 documented checkpoints with evidence of revision; process artifacts account for ≥20% of assessment weight.	Review submission logs for progressive revision; verify rubric includes process criteria.
Oral defense	Synchronous verification covers ≥25% of assessment weight; probing questions drawn from individual student’s process documentation.	Verify protocol includes student-specific probing; check oral defense is not generic.
Authentic tasks	Task requires context-specific data or real-time judgment not available to AI; a generic LLM prompt would be unlikely to produce a clearly passing response without task-specific human judgment.	Apply audit tool (Table 5); test by submitting task prompt to LLM and evaluating output.
AI-use policy	Policy specifies ILO-aligned AI-use levels with required disclosure; students receive policy before assessment.	Review assessment documentation for explicit policy; verify student receipt.

The proposed indicators and thresholds are heuristic design markers rather than validated cutoffs. They are intended to support comparative analysis and future empirical testing, not to function as universal minimum standards across disciplines.

## 5 A four-pillar framework for AI-resilient assessment

### 5.1 Derivation logic

**Figure 1:**
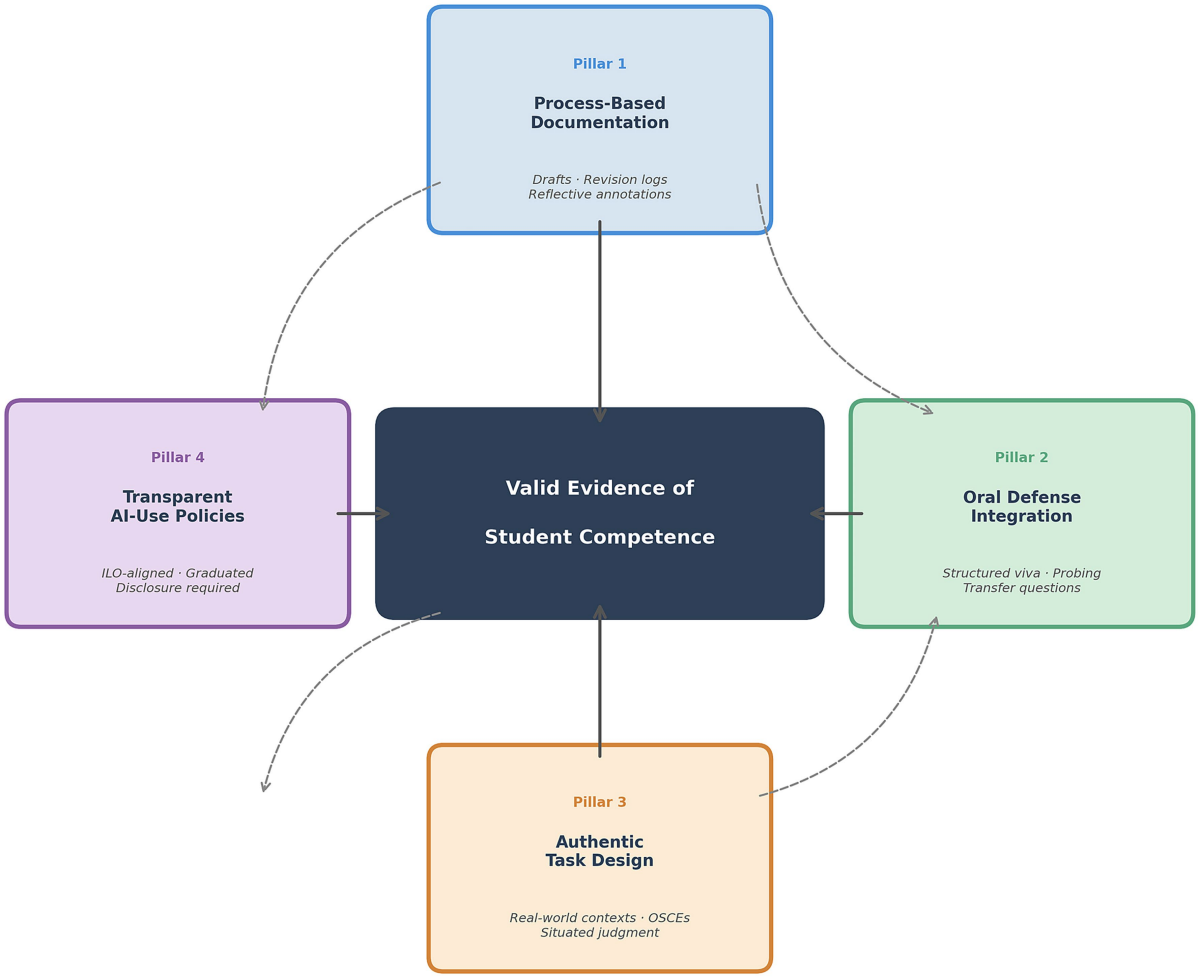
Four-pillar framework for AI-resilient assessment. Solid arrows: each pillar contributes a distinct type of evidence supporting competence inference. Dashed arrows: inter-pillar reinforcement (e.g., process documentation generates questions for oral defence). This diagram represents conceptual relationships, not empirically tested causal pathways.

The four pillars were derived from a structured conceptual analysis of the evidence base required to support valid inferences about student competence, drawing on Messick’s (1989) unified validity framework and constructive alignment (Biggs and Tang, 2011). Each pillar addresses a distinct evidentiary gap created by generative AI (see Section 3). The four pillars are not sub-constructs of competence; they are *design responses that secure evidence for competence inference in AI-rich contexts*. The central question is: what evidence architecture makes valid competence inference possible when students have access to generative AI? Figure 1 illustrates these relationships.

### 5.2 Pillar 1: process-based assessment

Process-based assessment shifts focus from the final product to the trajectory that produced it. By requiring drafts, revision logs, and reflective checkpoints, it creates documentation that is less easily outsourced to generative AI alone (Boud and Molloy, 2013). Process artifacts provide evidence of engagement, create reference points for oral defense, and encourage metacognition (Winne, 2018). However, process documentation should be interpreted as evidence of engagement, not proof of independent authorship, since process artifacts are not immune to fabrication.

### 5.3 Pillar 2: oral defense integration

Oral defense introduces a synchronous verification layer: students must demonstrate understanding during a live exchange, which is more likely to require direct student engagement than final-product submission alone (Joughin, 2010). The proposed protocol includes three phases: warm-up (student summarizes key conclusions, 2–3 min), probing (evaluator poses targeted questions from process documentation, 4–6 min), and extension (student applies reasoning to a novel scenario, 2–3 min). Total: 8–12 min per student.

### 5.4 Pillar 3: authentic task design

Authentic tasks require judgment under ambiguity and integration of multiple knowledge domains, making them less susceptible to generic AI outputs (Wiggins, 1990; Villarroel et al., 2018). In health sciences, these include simulated clinical encounters, case presentations with real-time questioning, and OSCEs (objective structured clinical examinations) integrating AI-generated data requiring human interpretation.

In engineering, authentic assessment might require students to design a load-bearing structure under material and cost constraints, present calculations to a panel, and defend trade-off decisions. In law, a simulated client interview followed by moot argument with spontaneous questioning requires real-time legal reasoning. In the humanities, students might curate primary sources for an exhibition and defend curatorial choices in seminar discussion.

Despite disciplinary differences, these examples share one design feature: students must justify decisions within a specific context and respond to follow-up challenge, rather than submit a polished product alone.

### 5.5 Pillar 4: transparent AI-use policies

Rather than blanket prohibitions, the framework advocates graduated policies aligned with intended learning outcomes (ILOs) (Lim et al., 2023). Table 4 presents a taxonomy. The central question is not whether AI is used, but whether its use obscures or preserves valid evidence of the targeted learning outcome.

## 6 Operationalizing the framework

This section presents the framework’s operational tools: a rubric model with worked scoring example, and an oral-defense protocol with workload analysis.

### 6.1 Rubric model

Table 3 presents a rubric model. Weight ranges reflect the framework’s design logic: reasoning and oral defense carry the largest share because they provide the strongest evidence of understanding that is less easily substituted by AI; process engagement documents the learning trajectory; product quality receives the smallest share as the dimension most susceptible to AI generation.

### 6.2 Oral-defense protocol and workload

The protocol comprises three phases (total 8–12 min per student). For a cohort of 100 students at 10 min each, the optimistic estimate is approximately 17 h of direct assessment time. Including preparation (reviewing process logs, ~5 min per student), scheduling, and moderation, a realistic range is 25–35 h per 100 students. For large programmes (300–600 students), full implementation requires substantial faculty coordination.

Random sampling—where all students prepare for oral defense but only a randomly selected subset (e.g., 30–40%) is assessed—reduces workload significantly but introduces a validity trade-off: the deterrence effect may weaken if students calculate low selection probabilities, and the framework’s evidentiary logic is only partially applied to unsampled students. Programs adopting random sampling should communicate clearly that selection is genuinely random and that any student may be selected, and should consider whether the remaining pillars (process documentation, authentic tasks) provide sufficient evidence for unsampled students. The validity implications of random sampling remain an open empirical question.

## 7 Illustrative applications

The scenarios below are hypothetical illustrations designed to show how the framework could be applied; they do not report actual implementation data.

### 7.1 Health sciences: surgical case analysis

In a surgical education module, a case analysis would be accompanied by a process log documenting the student’s initial differential diagnosis, prioritized clinical data, and reasoning evolution across two revision stages. The student then participates in oral defense: “You initially considered [Diagnosis A] but settled on [Diagnosis B]. Walk me through the clinical evidence.” Such questions require real-time clinical reasoning that cannot be adequately prepared through AI-generated text alone (Epstein, 2007).

### 7.2 Engineering: structural design project

In a second-year civil engineering module (n ≈ 200), students design a load-bearing structure under specified material, cost, and safety constraints. Under the framework: (1) Process documentation: students submit three staged deliverables—initial concept sketch with rationale, calculation spreadsheet with annotated assumptions, and final design report. (2) Oral defense: conducted in panels of 4–5 students with two assessors (approximately 15 min per student). For 200 students, direct oral assessment alone amounts to approximately 50 h of aggregate student-level assessment time before preparation and moderation are included. Running 8 panels in parallel reduces elapsed scheduling time, but not the total assessor workload. (3) Authentic task: site-specific data (soil reports, local building codes) are provided, preventing generic AI solutions. (4) AI-use policy: AI is permitted for initial literature search and code calculations (Guided level) but prohibited for the design rationale narrative.

This panel-based organization is presented as one feasible scaling strategy rather than a universal model; local implementation would depend on assessor availability, moderation procedures, and programme-level priorities.

## 8 Implications for multilingual and transnational contexts

Detection tools produce higher false-positive rates for non-native English writing (Liang et al., 2023), disproportionately flagging mobile students. A competency-focused approach may be more equitable. For transnational alliances (e.g., EUGLOH, Erasmus Mundus consortia), the four-pillar framework can serve as a shared vocabulary, and the policy taxonomy (Table 4) can harmonize AI-use expectations across partner institutions.

## 9 Implementation challenges

### 9.1 Faculty workload

The 17-h estimate per 100 students (Section 6.2) should be weighed against time currently spent on AI-detection investigations. Structured protocols, random sampling, and rubric-based marking can contain additional workload.

### 9.2 Student anxiety, accessibility, and equity

Oral assessment can provoke anxiety, particularly for students unaccustomed to verbal defense or facing language barriers (Huxham et al., 2012). The framework mitigates this through transparent criteria, advance notice of question types, and low-stakes practice.

However, oral defense as a heavily weighted criterion raises serious equity concerns for four groups: students with speech or hearing impairments; those with severe anxiety disorders or autism spectrum conditions; non-native speakers assessed in a second or third language; and students in fully online programmes without synchronous assessment capacity.

Three alternative verification formats are proposed, each targeting the same construct (demonstrated understanding and ownership) through a different modality:

- Written viva: The student receives individualized probing questions (drawn from their process documentation) and responds in writing under timed, supervised conditions. The same rubric criteria apply as for oral defense. This format removes oral fluency as a confound while preserving the requirement for real-time, student-specific reasoning.
- Asynchronous video defense: The student records video responses to a set of probing questions, followed by a written follow-up round addressing evaluator queries. Suitable for fully online programmes where synchronous scheduling is impractical.
- Extended portfolio verification: Process documentation is expanded to include detailed reflective annotations at each checkpoint, with explicit justification of decisions. The portfolio is evaluated against the oral-defense rubric criteria, with the reflective annotations serving as a written equivalent of oral probing.

The principle of equivalence is essential: alternative formats should be evaluated against comparable criteria, differing only in modality. Establishing empirical equivalence between oral and alternative formats is itself a research question requiring future investigation. Programs should document which format each student uses and monitor for differential outcomes across formats.

The equity paradox should be acknowledged explicitly: oral defense may reduce one form of bias (AI-detection false positives for non-native writers) while introducing another (oral assessment disadvantage for the same population). This tension cannot be fully resolved within the framework alone and requires ongoing monitoring.

### 9.3 Disciplinary variation

Table 5 provides a vulnerability audit tool.

### 9.4 Boundary conditions and implementation contexts

The framework is most directly applicable in disciplines that assess explanation, judgment, and context-sensitive reasoning—particularly professional and practice-oriented programmes. Three implementation scenarios illustrate how feasibility varies by context:

- Large cohort (*n* = 300, health sciences): All four pillars can be implemented if oral defense uses random sampling (30–40% of students selected per cycle, with all students informed they may be selected). Process documentation and AI-use policy apply to all students. Estimated additional faculty time: ~50 h per assessment cycle (including preparation and moderation for sampled students). The validity trade-off of random sampling must be acknowledged: unsampled students are assessed on three pillars only.
- Resource-constrained institution: Pillars 1 (process documentation), 3 (authentic tasks), and 4 (AI-use policy) can be implemented with minimal additional infrastructure—primarily requiring version-tracked submission platforms and clear policy communication. Pillar 2 (oral defense) can be introduced selectively for capstone or high-stakes assessments only.
- Fully online/asynchronous programme: Oral defense can be replaced with written viva or asynchronous video defense (see Section 9.2). Process documentation works natively in digital environments. Authentic tasks may require adaptation to use online-accessible but student-specific data sets.

The framework should be understood as a modular set of design principles. Partial implementation—adopting two or three pillars where full implementation is infeasible—is preferable to maintaining AI-vulnerable assessment unchanged, provided the limitations of partial coverage are acknowledged.

### 9.5 Framework vulnerability to evolving AI capabilities

The framework is proposed as AI-resilient, not AI-proof, and its own components may become vulnerable as AI capabilities evolve. Process documentation (Pillar 1) could be fabricated using AI-generated revision histories or staged drafts on collaborative platforms. Oral defense (Pillar 2) may become vulnerable to real-time AI coaching via earpieces, screen-based prompts, or future AI-mediated conversational agents. Authentic task design (Pillar 3) may be partially undermined as multimodal AI systems improve at situated reasoning. AI-use policies (Pillar 4) face enforcement challenges as AI tools become more seamlessly integrated into student workflows.

Second-order safeguards can mitigate some of these risks: timestamped platform-based drafting (reducing the credibility of fabricated process logs); proctored or in-person oral defense (reducing AI-coaching risk); tasks incorporating local, unpublished, or real-time data (limiting pre-preparation with AI); and institutional monitoring of AI-tool evolution to update policies. However, no assessment design is permanently invulnerable, and ongoing adaptation will be required. The framework’s principal defence is its multi-layered architecture: circumventing any single pillar is insufficient if the remaining pillars still generate valid evidence of competence.

## 10 Discussion and conclusion

The framework redirects assessment design from verifying who wrote the submitted text to evaluating whether the student can demonstrate the targeted competency through explanation, application, and real-time reasoning. This shift aligns with constructivist learning theory: assessments capturing only the endpoint miss the iterative process through which understanding develops (Vygotsky, 1978; Biggs and Tang, 2011). It may also advance equity by providing multiple modalities for demonstrating competence.

Written products remain valuable, but they should function as one component within a broader evidence base that also captures reasoning processes, oral justification, and applied judgment. In this sense, the framework translates concerns about AI-vulnerable assessment into concrete design choices (Bearman et al., 2023; Corbin et al., 2025a).

Several limitations should be acknowledged. The framework has not been empirically validated; controlled studies comparing outcomes under traditional and AI-resilient formats are needed. The proposed tools have not been evaluated for reliability, inter-rater consistency, or transferability. Oral defense creates accessibility challenges (Section 9.2) and may introduce linguistic biases. Illustrative examples are drawn primarily from health sciences and engineering; cross-disciplinary validation is needed.

Beyond these technical limitations, three structural barriers may affect adoption. Institutionally, implementing oral defense and process-based assessment may require changes to examination regulations, quality-assurance frameworks, and workload allocation models; without institutional leadership support and faculty development programmes, adoption is unlikely to proceed beyond individual enthusiasts. Pedagogically, oral defense may shift assessment culture toward performance rather than understanding; training assessors to conduct consistent, fair oral examinations is a non-trivial undertaking; and process documentation requirements may encourage strategic compliance (producing staged drafts to satisfy requirements) rather than genuine reflection. Culturally, assessment traditions vary across educational systems—oral examination is central in some continental European traditions but peripheral in others; power dynamics in oral defense contexts may disadvantage students from cultures where challenging authority is discouraged; and multilingual settings require sensitivity to the difference between linguistic fluency and conceptual understanding.

The framework addresses assessment design but does not, on its own, resolve the broader organizational, pedagogical, and cultural challenges of AI integration in higher education. It should be viewed as one component of a larger institutional response.

Future empirical work should include: inter-rater reliability for rubric and oral-defense scoring (using intraclass correlation coefficients or weighted kappa); feasibility metrics (time per student, faculty acceptability, completion rates); differential impact analysis by language status and disability; response-process evidence examining how students and assessors interact with the tools; and pilot comparisons with conventional assessments.

A concrete first step would be a quasi-experimental pilot: in a single health sciences course (n ≥ 60), one cohort submits a traditional written case analysis while a parallel cohort completes the same task under the four-pillar format (process log + oral defense + authentic scenario + declared AI-use policy). Primary outcomes would be inter-rater reliability for the rubric (target ICC ≥ 0.70, requiring approximately 50 student submissions rated by two independent assessors) and student-reported acceptability. Secondary outcomes would include faculty time per student and proportion of students whose oral-defense performance diverged substantially from their written-product grade. Such a study would provide the initial feasibility and validity evidence needed to move the framework from conceptual proposal toward evidence-based practice. A further priority would be evaluating the empirical equivalence of the alternative verification formats proposed in Section 9.2 (written viva, asynchronous video defense, extended portfolio verification) for students with disabilities or those in fully online programmes. Such a study could compare scores, inter-rater reliability, and student-reported fairness across oral and alternative formats, using a within-subjects or matched-pairs design, to determine whether these alternatives provide comparable evidence of understanding and ownership.

The proliferation of generative AI compels a structural re-evaluation of which evidence warrants competence inferences and how assessment architectures can secure that evidence. The framework and tools presented here are intended as practical starting points for local adaptation and empirical evaluation—not as final solutions, but as a structured foundation on which both institutional practice and future research can build.
